# Supplementary material for: Flexible Pressure Sensors Based on P(VDF-TrFE) Films Incorporated with Ag@PDA@PZT Particles
Source: Sensors (Basel). 2024 Aug 21;24(16):5415. doi: 10.3390/s24165415 (PMC11359586; doi:10.3390/s24165415)
Supplement: Supplementary file 1 [file sensors-24-05415-s001.zip › sensors-3153876-supplementary.pdf]

Supporting Information

Flexible Pressure Sensors Based on P(VDF-TrFE) Films Incorporated with Ag@PDA@PZT Particles

Yingzheng Mei, Chuan Cao, Peng Zhou *, Jianqiao Wang, Miaoxuan Liu, Xunzhong Shang, Juan Jiang, Yajun Qi and Tianjin Zhang *

Ministry of Education Key Laboratory for Green Preparation and Application of Functional Materials, Hubei Provincial Key Laboratory of Polymers, Collaborative Innovation Center for Advanced Organic Chemical Materials Co-Constructed by the Province and Ministry, School of Materials Science and Engineering, Hubei University, Wuhan 430062, China

* Correspondence: zhou@hubu.edu.cn (P.Z.); zhangtj@hubu.edu.cn (T.Z.)

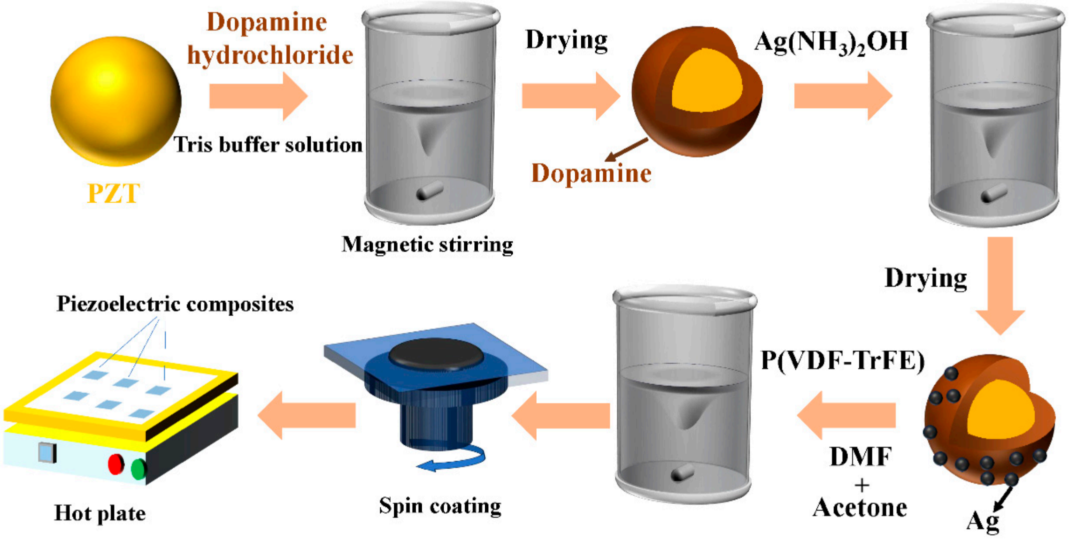

Figure S1 Schematic diagram of sensor preparation process

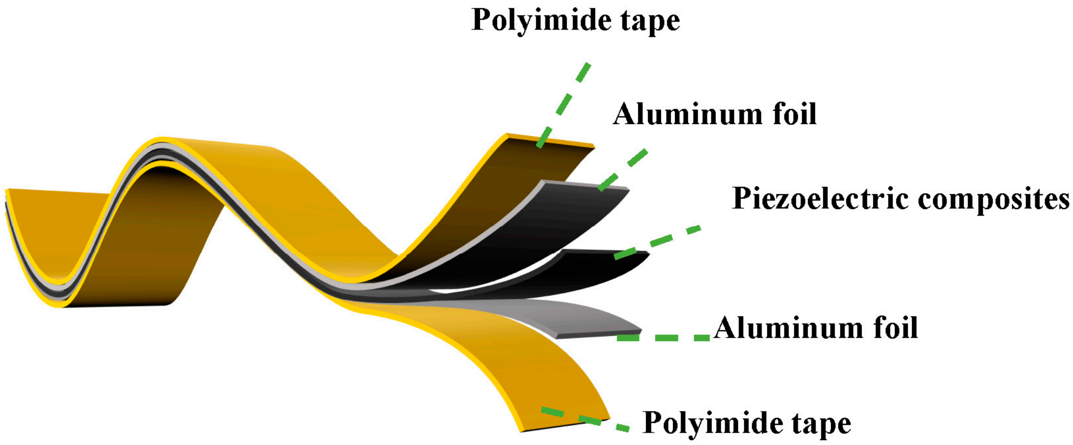

Figure S2 Schematic diagram of sensor structure

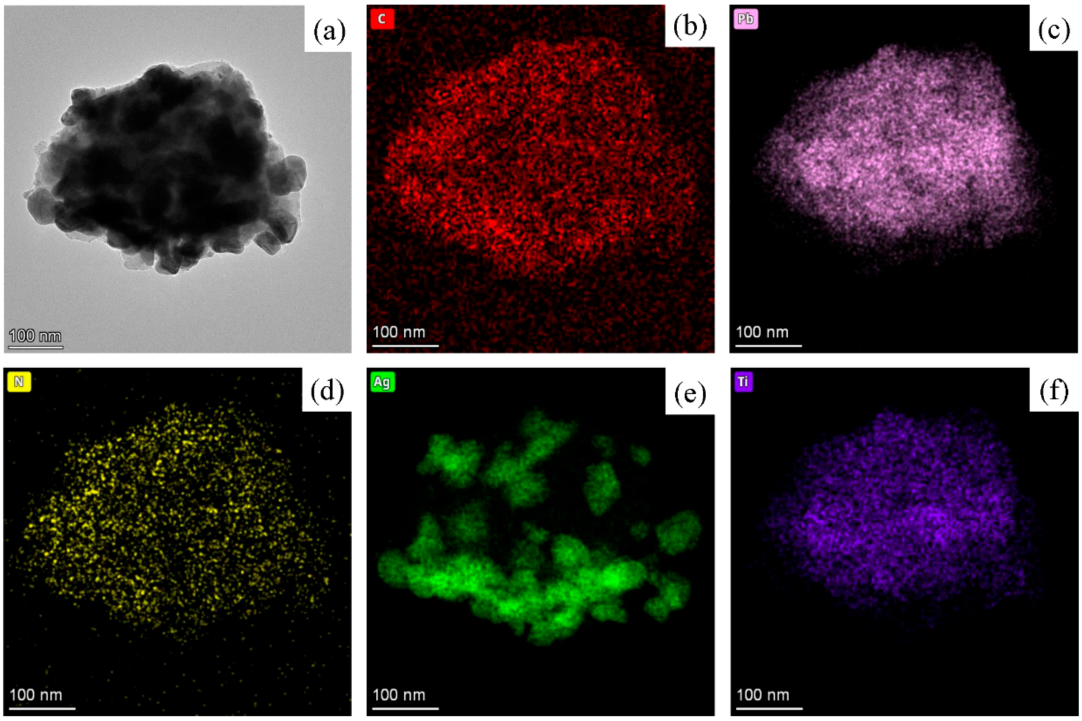

Figure S3 TEM images of Ag@PDA@PZT particle, (a) topography, (b) – (f) EDS mapping for C, Pb, N, Ag, and Ti, respectively

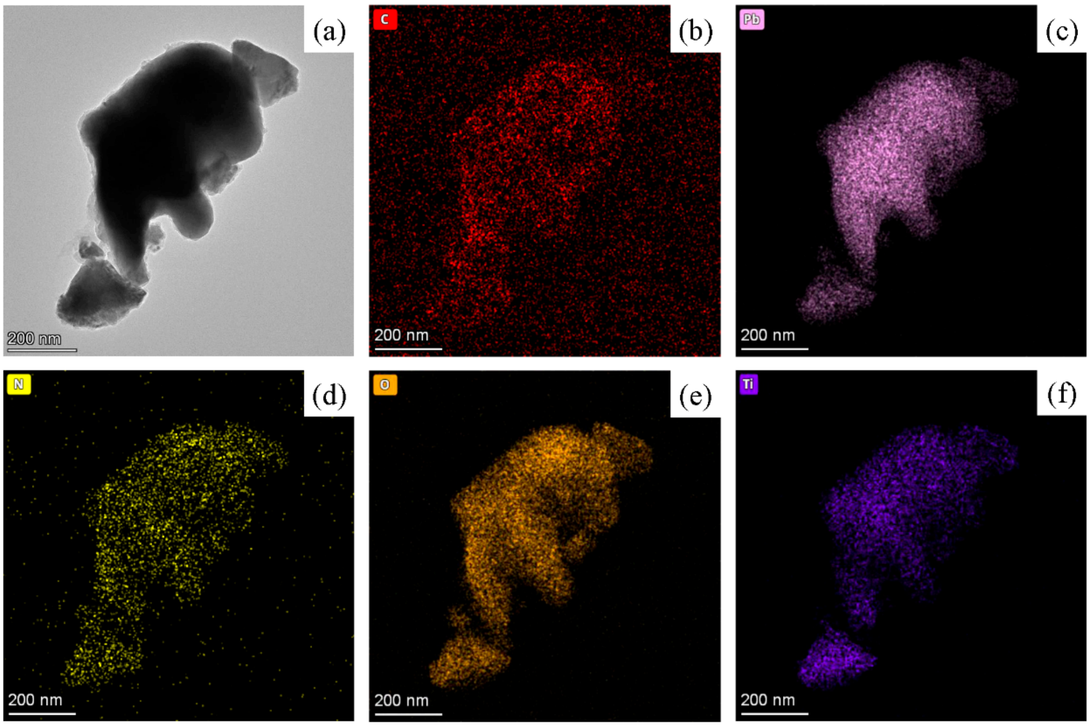

Figure S4 TEM images of PDA@PZT particle, (a) topography, (b) – (f) EDS mapping for C, Pb, N, O, and Ti, respectively

Piezoelectric response mechanism of composites:

Piezoelectric response mechanism of the composites is illustrated in figure S5. The dipoles in piezoelectric composite exhibit random orientation before poling, as shown in figure S5(a). After poling, most of the dipoles align along the direction of external electric field. Opposite charges accumulate on both electrodes to shield the internal electric field, as shown in figure S5(b). When external force is applied, a potential difference is generated due to the reorientation of dipoles in the piezoelectric composites. Then the charges migrate and accumulate on both electrodes to shield the induced piezoelectric potential. Hence, the directional movement of the charges gives rise to the observed current, as shown in figure S5(c). After removal of the external force, the charges flow along the opposite direction, resulting in a reverse current, as shown in figure S5(d). Therefore, the pressure sensor generates continuous alternating electrical signals under constant force. More net dipole moments can generate higher potential difference under the same force.

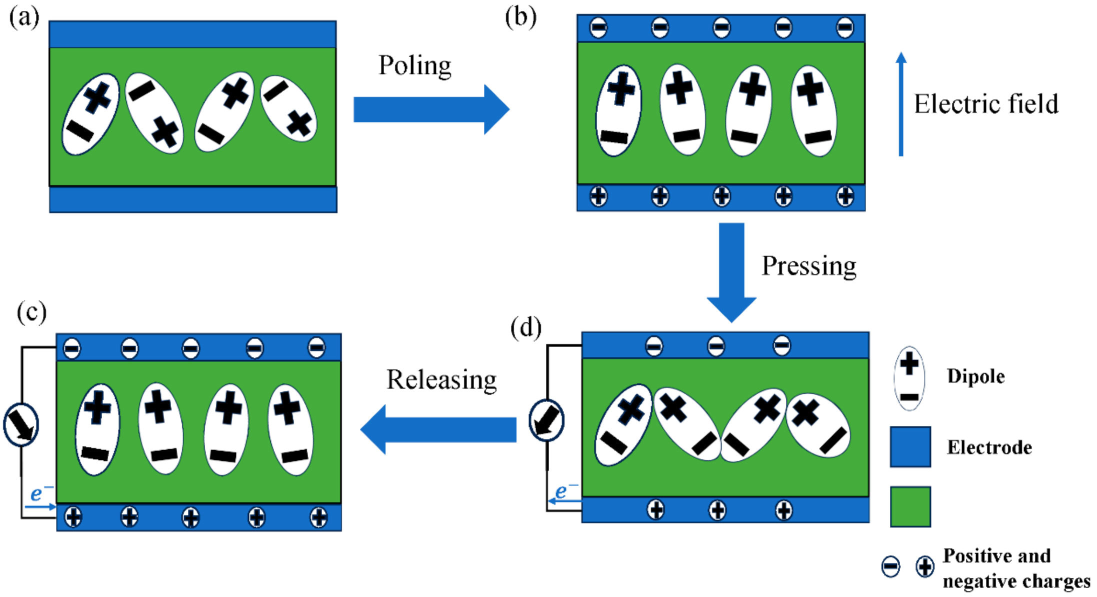

Figure S5 Schematic diagram of piezoelectric response mechanism of composites

Simulation parameters:

COMSOL Multiphysics simulation (the finite element analysis) was employed to simulate the output electric potential distribution of piezoelectric composites under strain. The three-dimensional model with length, width, height of 3 μm, 3 μm, 1 μm, was established. The radii of Ag, PZT, and PDA@PZT were 20 nm, 200 nm, and 280 nm, respectively. The applied strain in piezoelectric composites was fixed to 10%.
